# Microbiota Associated With *Ototyphlonemertes* Species (Nemertea, Hoplonemertea, Monostilifera, Ototyphlonemertidae) Reveal Evidence of Phylosymbiosis

**DOI:** 10.1002/ece3.70471

**Published:** 2024-12-03

**Authors:** Francesca Leasi, Ester M. Eckert, Jon L. Norenburg, W. Kelley Thomas, Joseph L. Sevigny, Jeffrey A. Hall, Herman H. Wirshing, Diego Fontaneto

**Affiliations:** ^1^ Department of Biology, Geology, and Environmental Science University of Tennessee at Chattanooga Chattanooga Tennessee USA; ^2^ National Research Council of Italy (CNR), water Research Institute (IRSA) Molecular Ecology Group (MEG) Verbania Pallanza Italy; ^3^ National Museum of Natural History Smithsonian Institution Washington, DC USA; ^4^ Hubbard Center for Genome Studies University of New Hampshire Durham New Hampshire USA

**Keywords:** meiofauna, microbiome, microbiota, Nemertea, phylosymbiosis

## Abstract

Phylosymbiosis, the association between the phylogenetic relatedness of hosts and the composition of their microbial communities, is a widespread phenomenon in diverse animal taxa. However, the generality of the existence of such a pattern has been questioned in many animals across the tree of life, including small‐sized aquatic invertebrates. This study aims to investigate the microbial communities associated with poorly known marine interstitial nemerteans to uncover their microbiota diversity and assess the occurrence of phylosymbiosis. Specimens from various Central American sites were analyzed using morphology‐based taxonomy and molecular techniques targeting the host 18S rRNA gene whereas their microbial association was analyzed by targeting the prokaryotic 16S rRNA gene. Phylogenetic and statistical analyses were conducted to examine the potential effects of host nemertean taxa and sampling locations on the host‐associated microbial communities. The results provide compelling evidence of phylosymbiosis in meiofaunal nemertean species, highlighting the significant impact of host genetic relatedness on microbiome diversity in small‐sized animals. This finding supports previous studies that demonstrate how certain nemertean species harbor distinct microbial communities with functional and ecological implications. Given the remarkable diversity of meiofaunal animals—spanning numerous phyla with varying lifestyles and co‐existing in the same habitat—combined with advancements in multi‐omics approaches, there is a promising opportunity to deepen our understanding of the evolutionary and ecological interactions between hosts and their microbiota throughout the animal tree of life.

## Graphical abstract

This study explores phylosymbiosis, the connection between host phylogenetic relatedness and microbial community composition, in marine interstitial nemerteans. The research reveals compelling evidence of phylosymbiosis in these small aquatic animals, emphasizing the influence of host genetic relatedness on microbiome diversity.

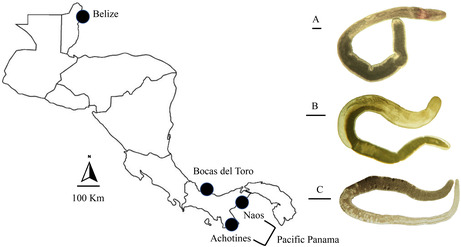

## 1 Introduction

Phylosymbiosis elucidates the interconnections between the ecological and evolutionary relationships of host organisms and the composition of their associated microbial communities, called microbiota. This pattern arises when a significant correlation is observed between host phylogenies and microbiota composition, suggesting that microbial communities are shaped by the host's evolutionary history, biological traits, and ecological factors, such as dietary niches (Erlandson et al. 2018; Grieneisen et al. 2019; Ley et al. 2008; Lim and Bordenstein 2020; Yeoh et al. 2017).

Over the past decade, there has been a significant expansion in research exploring the relationship between hosts and their microbiota. As a result, investigations into microbiota have extended beyond humans to encompass other vertebrates, plants, invertebrates, and protists (Ahern et al. 2021; Akyol, Sato, and Turkan 2020; Gomaa et al. 2022; van Oppen and Blackall 2019; Vernier et al. 2020). Across the animal kingdom, research on host‐associated microbiota have provided compelling evidence for the widespread prevalence of phylosymbiosis. Numerous animals harbor microbial symbionts that fulfill crucial roles in their health and vital functions (Dalenberg et al. 2020; Röthig et al. 2016; Youngblut et al. 2019). These symbiont microorganisms can significantly influence host development, immunity, and metabolism (Conroy and Holman 2022; Dalenberg et al. 2020; Malešević et al. 2021; Miller et al. 2023) while also facilitating ecological interactions and contributing to the emergence and diversification of animal groups (Archie and Tung 2015; Douglas 2018; Vernier et al. 2020; Zilber‐Rosenberg and Rosenberg 2008). However, most studies investigating patterns of phylosymbiosis have predominantly focused on only a small fraction of animal lineages such as terrestrial and marine mammals and fish (Apprill et al. 2020; Brown et al. 2023; Ley et al. 2008; Moeller et al. 2017; Sadeghi et al. 2022), terrestrial arthropods, particularly spiders and insects (Dunaj et al. 2020; Li et al. 2022; Tinker and Ottesen 2020), or, in the marine environment, corals and sponges (Moitinho‐Silva et al. 2017; Pushpakumara et al. 2023; Röthig et al. 2016; Sweet and Bulling 2017; Thomas et al. 2016). Other organisms residing in various ecosystems and distinguished by a range of life histories have received relatively less attention in comparison. Expanding the scope of research to encompass these organisms provided a more comprehensive understanding of host‐microbiota associations across diverse biological systems, ecological features, and evolutionary clades (McFall‐Ngai et al. 2013; Zilber‐Rosenberg and Rosenberg 2008).

Recent studies exploring the microbiota associated with microscopic aquatic invertebrates have revealed a notable absence or limited presence of phylosymbiosis (Boscaro et al. 2022). In contrast to many larger animals, the microbiota composition in most microscopic aquatic organisms across different phyla, whether freshwater or marine, exhibits remarkable flexibility, largely shaped by the surrounding environment (Boscaro et al. 2022; Eckert, Anicic, and Fontaneto 2021; Rosa and Loreto 2022; Schuelke et al. 2018; Turgay et al. 2020). The absence of phylosymbiosis is not exclusive to microscopic aquatic invertebrates, as exemplified by a study on larger terrestrial arthropods, such as caterpillars, which demonstrated that these insects benefit from ecological and evolutionary independence from symbionts (Hammer et al. 2017). These findings are particularly intriguing, considering that caterpillars often represent the sole feeding stage in lepidopterans. Also, among vertebrates across various habitats, such as in fishes, amphibians, and terrestrial mammals, host‐microbiota associations can be driven more by the ecomorphology of the hosts rather than by their phylogenetic relationships (Bletz et al. 2017; Escalas et al. 2021; Grond et al. 2020).

In conclusion, significant heterogeneity exists among host‐microbiota associations, making it difficult to generalize patterns across different species or habitats. In fact, some animals may not even require a microbiota at all (Hammer, Sanders, and Fierer 2019). Therefore, it is essential to take into account the unique biological and ecological characteristics of each host organism and its associated microbiota to gain a comprehensive understanding of their relationship.

The aim of this study is to investigate the patterns in the diversity and potential phylosymbiosis in the community composition of microbes associated with interstitial marine nemerteans. Nemertea, known also as ribbon worms or proboscis worms, are marine worm‐shaped animals that have received relatively limited attention in research due to the combination of restricted expertise and the deceptive morphological complexity exhibited by their species (Leasi and Norenburg 2014). Nevertheless, certain nemertean species, such as the invasive *Cephalothrix simula*, have been found to harbor distinct microbial communities with functional and ecological implications, including high toxicity (Turner et al. 2018). Overall, *Vibrio* spp. were identified as the most common bacteria associated with nemerteans. Along with *Alteromonas* spp. and a species of *Bacillus*, these bacteria were found responsible for producing tetrodotoxin, a potent toxin found in marine species, which likely aids in both predation and defense against predators (Turner et al. 2018). Several nemertean taxa representing Tetrodotoxin‐bearing and non‐Tetrodotoxin‐bearing species also demonstrated a strong association between nemertean species and their associated microbiome (Melnikova and Magarlamov 2020). Although evidence suggests that nemertean species may exhibit a species‐specific relationship with their microbiota, whether beneficial, detrimental, or neutral, the extent to which the microbial composition can be attributed to the phylogenetic relationship of the host remains less understood.

This research seeks to expand upon the taxonomic breadth of previous investigations on phylosymbiosis in small‐sized organisms with the specific objective of untangling the role of host evolutionary history in elucidating variations in microbiomes associated with a group of marine interstitial microscopic nemerteans.

## 2 Material and Methods

### 2.1 Sampling

Adult specimens were collected from sites visited during four biodiversity workshops held in Central America in 2010, 2011, and 2016, focused on marine meiobenthos, namely the community of microscopic animals living in the marine sediments (Giere 2009). The collection of samples took place at specific sediment sites during different periods: (i) Carrie Bow Cay, Belize (Atlantic Ocean) in June 2010; (ii) Bocas del Toro, Panama (Atlantic Ocean) in October 2010; and (iii) in the Pacific side of Panama, namely at Naos Island in December 2011 as well as Achotines Bay in February 2016. A total of 230 nemertean specimens were collected and stored for DNA analyses. Collecting permits were issued by James Azueta, Comptroller of Customs, Belize Fisheries Department, Ministry of Agriculture & Fisheries to FL or JLN: Ref: GEN/FIS/15/04/10–52, Vol. III; Mario Quirós, Director General, Ecargado, Autoridad de los Recursos Acuáticos de Panama: Resolución DGOMI‐PICFC‐No. 40 de 31 Octubre de 2011, and Samuel Valdés Díaz, Director de Áreas Protegidas y Vida Silvestre respectively (Permiso Científico No SE/A‐2‐16; January 4, 2016). No endangered or protected species were involved in any of our work. Meiofaunal animals were extracted from the sediment using magnesium chloride isotonic to seawater; those ones belonging to the phylum Nemertea were isolated, identified to the lowest practical taxon rank, rinsed in clean water for a few minutes, and transferred to 70% ethanol in DNA barcode tubes marked with unique extraction barcodes.

### 2.2 DNA Extraction

The extraction of genomic DNA from each individual nemertean was carried out at the Laboratories of Analytical Biology (LAB) at the Smithsonian Institution National Museum of Natural History (NMNH) in Washington, D.C. (USA), using an automated DNA extraction system (AutoGen, Inc) and standardized protocols. This method was previously described in other studies involving certain nemertean specimens included in the present investigation (Leasi, Andrade, and Norenburg 2016; Leasi et al. 2018). Tissue digestion was performed at 56°C in a shaker incubator with 150 μL of M2 buffer mixed to 150 μL of M1 buffer and Proteinase K. DNA extraction was performed using the Autogen Prep 956 Extractor. The DNA was eluted in 100 μL of R9 buffer and is currently stored at the NMNH biorepository in tubes marked with unique barcodes. Manufacturer buffers were provided by Autogen, Inc.

### 2.3 DNA Amplification and Sequencing

Two separated polymerase chain reactions (PCRs) were performed on the genomic DNA of each single individual nemertean to retrieve (1) the eukaryotic V1–V2 region of 18S rRNA gene of the animal using a Sanger‐based sequencing approach for the host, and (2) the prokaryotic V4 region of 16S rRNA gene using a DNA amplicon‐based sequencing approach (DNA metabarcoding) for the associated microbiota.

1. To retrieve the eukaryotic nuclear gene 18S rRNA gene, PCR was carried out at LAB (NMNH) using the 18S EukF forward primer (Sands et al. 2008) [AACCTGGTTGATCCTGCCAGT] and the SR7 reverse primer (Vilgalys and Sun 1994) [GTTCAACTACGAGCTTTTTAA]. A 25 μL final volume with 50 mM Tris–HCl pH 9.1, 16 mM (NH_4_)2SO_4_, 3.5 mM MgCl_2_, 150 mg/mL bovine serum albumin (BSA), 0.5 mM of each primer, 160 mM of each dNTP, and 0.05 U/μL of KlenTaq polymerase (AB Peptides, Inc.) with manufacturer provided buffers was the input to the thermal cycler. Thermal cycler parameters comprised an initial 3‐min denaturation at 95°C, followed by 35 cycles of 30 s at 95°C, 30 s at 52°C, 45 s at 72°C. The cycling ended with a 7‐min sequence extension at 72°C. PCR products were purified with Illustra Sephadex columns and then used for cycle sequencing with dye‐terminators using BigDye chemistry (Perkin‐Elmer) and standard cycling (4 min denaturation at 96°C, followed by 25 cycles of 10 s at 96°C, 5 s at 50°C, and 4 min at 60°C). Cycle‐sequenced products were run on an ABI 3730xl 96‐well capillary sequencer at LAB (NMNH).
2. DNA amplification, purification, and sequencing for metabarcoding analyses were all conducted at the Hubbard Center for Genomic Studies (HCGS) at the University of New Hampshire in Durham NH (USA), following the Earth Microbiome Project (earthmicrobiome.org) protocol for 16S rRNA gene (region V4) Illumina Amplicon (Caporaso et al. 2010). Specifically, the 16S rRNA gene (region V4) was amplified using Illumina_515f forward primer (Parada, Needham, and Fuhrman 2016) [GTGYCAGCMGCCGCGGTAA] and Illumina_926R reverse primer (Parada, Needham, and Fuhrman 2016) [CCGYCAATTYMTTTRAGTTT]. The input to the thermal cycler was a final volume of 25 μL including 12 μL of PCR‐grade water, 10 μL of PCR master mix, 1 μL of forward primer (5 μM), 1 μL of reverse primer (5 μM), and 1 μL of template DNA. Thermal cycler conditions included an initial denaturing step of 94°C for 3 min, followed by 35 cycles of 94°C for 45 s, 50°C for 60 s, 72°C for 90 s, and a final extension step of 72°C for 10 min. Each round of amplification included a negative (PCR‐grade water) control. Purification and sequencing proceeded only when gel electrophoresis did not show evidence of contamination in the negative control. The PCR products were purified using the Rapid PCR Purification System Kit (Marligen Bioscience, Inc.) and further sequenced on an Illumina HiSeq2500 at HCGS.

### 2.4 Computational Analyses

#### 2.4.1 Host Phylogeny Based on Eukaryotic 18S rRNA Gene

Forward and reverse partial 18S rRNA gene sequences obtained from single organisms were merged and each complete sequence checked and edited in Geneious Prime version 2022.1.1 (www.geneious.com) in case mismatches were present. Complete sequences of about 600 bp long (18S rRNA gene, region V1 and V2) were checked for possible contaminations or misidentifications using BLAST. An alignment of 18S sequences from the nemertean hosts was obtained using Q‐INS‐i algorithm in MAFFT v 7.0 (Katoh and Standley 2013) to account for the secondary structure of ribosomal DNA. No outgroup was used, and the tree was rooted using the main subdivision in groups of Nemertea. Given the current ambiguity in higher divisions in Nemertea (Chernyshev 2021), we kept Hoplonemertea as monophyletic, separated from a clade with the other groups, Palaeonemertea and Pilidiophora. The phylogeny was not used to reconstruct speciation events, but only to represent relationships between individuals, to be compared with the differences in their microbiome. We performed a maximum likelihood reconstruction using PhyML 3.0 with GTR substitution model (Guindon et al. 2010) and then made the tree ultrametric in the R package *ape* v5.0 (Paradis and Schliep 2019).

#### 2.4.2 Associate Microbiome Based on Prokaryotic 16S rRNA Gene

After primers and adapters removal, the raw genetic reads were imported into QIIME2 platform version 2021.8 (Bolyen et al. 2019) for sequence analyses and processing. Further sequence quality control and generation of amplicon sequence variants (ASVs) were completed using the DADA2 V1.4 pipeline (Callahan et al. 2016). This was accomplished by removing nucleotides from both the forward and reverse reads up to position 7 and from position 220. Therefore, paired‐end reads were merged and after chimera checking and denoising, we obtained a total of 679,705 reads clustered into 4627 ASVs with average length of 352 base pairs (bp). The taxonomy of each ASV was determined in QIIME2 by comparing the top hit identified with BLAST against the SILVA database number 99, version 138 (Quast et al. 2012). Following an examination of taxonomic assignments for each ASV, further quality control steps were undertaken. These steps encompassed the elimination of off‐target ASVs, such as eukaryotic genetic sequences, including sequences linked to chloroplasts and mitochondria, as well as unassigned ASVs. Given that microbiomes were obtained from animals, a large part of the amplified reads had to be discarded due to non‐specific primer binding and consequent amplification of animal DNA. The dataset was further reduced in order to keep only the animals for which both 18S rRNA gene and microbiome data were available and with microbiome represented by at least 1000 reads per individual nemertean specimen, a measure taken to prevent issues related to low read numbers potentially underestimating diversity. Because of these reasons, a low success rate, expected when working with single microscopic animals like meiofaunal nemerteans, led to a final reliable dataset consisting of 55 nemertean hosts and 1393 16S rRNA ASVs from the associated microbiomes. Read abundances were then summarized on a prokaryotic genus level and plotted per nemertean species. A graphical representation of frequency of microbial genera across nemertean species was limited to the genera with more than 1000 reads in the total dataset and that occurred in at least 3 samples. Data processing was completed using the R v4.1.3 (R Developement Core Team 2022) packages *stringr* v. 1.5.0 (Wickham 2019) and *dplyr* 1.0.9 (Wickham 2019) whereas *ggplot2* 3.4.0 (Wickham and Sievert 2016) and *ggtext* 0.1.2 (Wilke and Wiernik 2022) were used for the graphs.

### 2.5 Hypothesis Testing

We tested whether the host identity could influence the community composition of the microbiome. The first hypothesis we tested was whether each host taxon could have its own distinct microbiome. Differences in microbiome between samples were measured as Bray–Curtis abundance‐based dissimilarities in the R package *vegan* v2.5–7 (Oksanen et al. 2022). We started at higher taxonomic rank by testing whether different genera of Nemertea could host different microbiomes using a Permutational Analysis of Variance (PERMANOVA) with the matrix of Bray–Curtis pairwise dissimilarities in microbiomes as the response variable and host genus identity as an explanatory variable. Given that samples originated from three main geographic areas (Belize, Bocas del Toro, and Pacific Panama), the identity of the geographic area was also included in the model as an additional explanatory variable, accounting for geography as a potential additional predictor of microbiome composition. Then, focusing only on the most densely sampled genus, *Ototyphlonemertes*, a PERMANOVA was used to assess the role of species identity and geographic area of origin in explaining differences in microbiome composition. PERMANOVAs were performed with R package *vegan* and, for the genus *Ototyphlonemertes*, visualized as non‐metric multidimensional scaling (nMDS) and hierarchical clusters with average agglomeration method, with plots obtained with the R package *ggplot2*.

To test the correlation between the phylogenetic relationships of the hosts and their microbiomes, we performed a Mantel test between the matrix of Bray–Curtis distances in microbiome composition for each individual and the matrix of patristic distances of the same individual nemertean from the phylogenetic tree of the hosts, obtained with the R package *ape*. In addition, to account for the confounding effect of geographical distances, we performed partial Mantel tests between the Bray–Curtis distances in microbiome composition and the patristic distances of the phylogenetic tree of the hosts, controlling for the matrix of geographical distances between the samples. Geographical distances were calculated both linearly as the crow flies and following the coastline. Geographical distances were obtained using the R package *marmap* v1.0.10 (Pante et al. 2023), and Mantel tests were performed with the R package *vegan*.

## 3 Results

**FIGURE 1:**
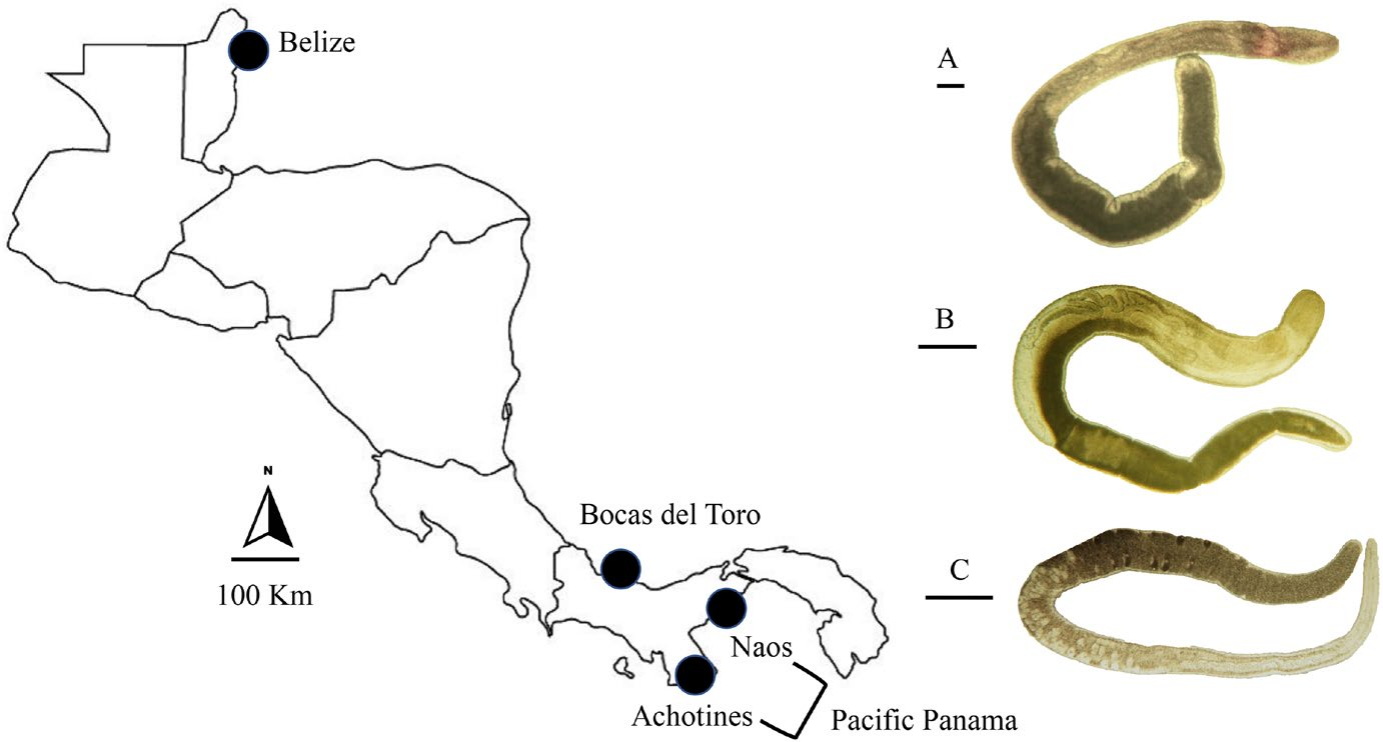
Representatives of nemertean representatives used in this study and map showing collection sites. A. *Ototyphlonemertes macintoshi* found in Belize; B. *Annulonemertes* sp. found at Bocas del Toro; and C. *Cephalothrix* sp. found at Bocas del Toro and in Pacific Panama. Scale bars = 100 μm.

**FIGURE 2:**
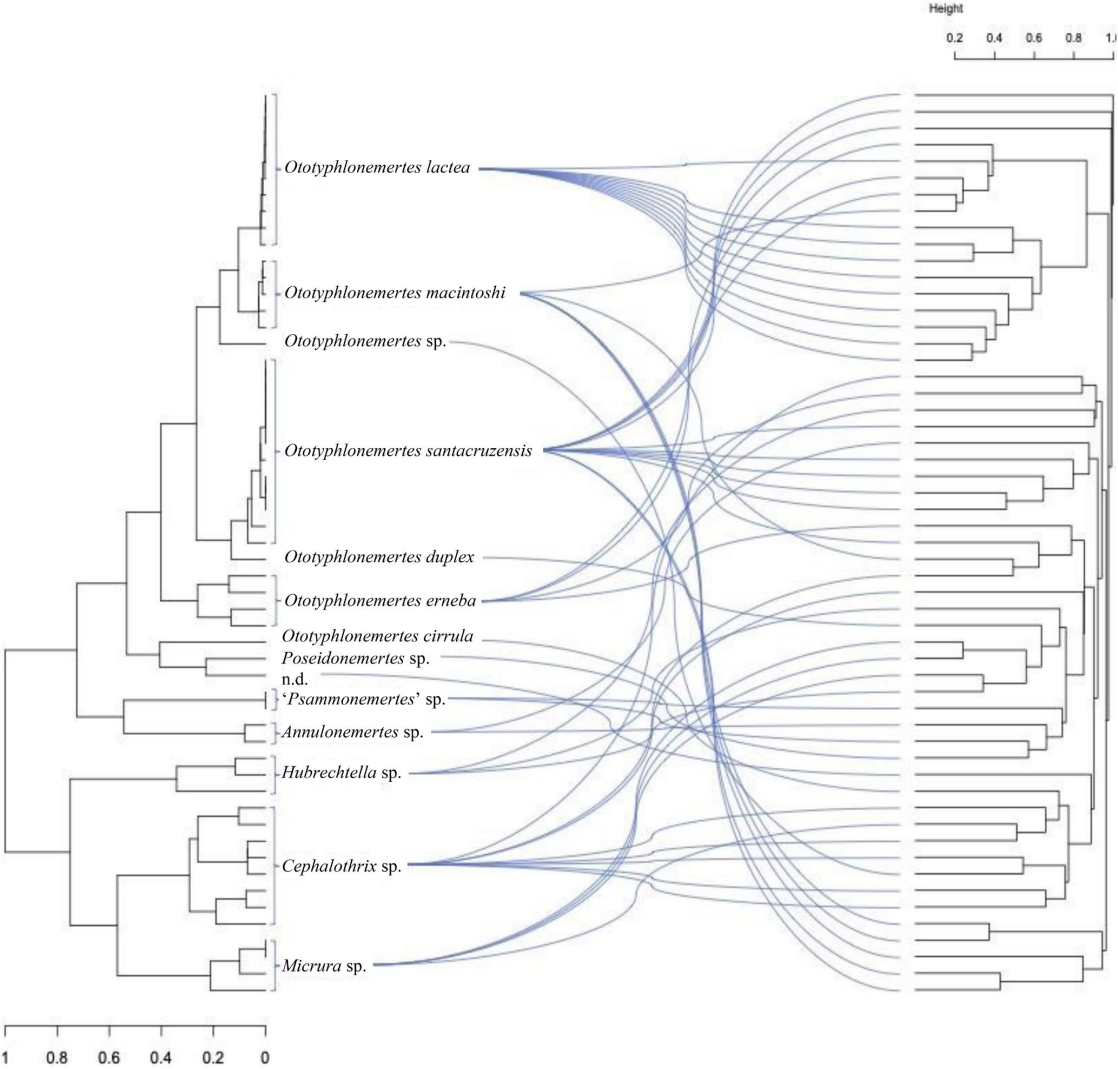
Tanglegram of host‐microbiome relationships. The host phylogeny on the left is a Maximum Likelihood tree with branch length and scale bar proportional to evolutionary rates in an 18S rRNA gene alignment. The cladogram on the right represents a clustering of Bray‐Curtis dissimilarities in microbial composition between samples, where each clade represents a microbial community present in one nemertean specimen.

The study encompassed an examination of 55 nemertean specimens, categorized into six distinct families, six identified genera, and a minimum of 14 morphological species (Table 1; Figure 1). Among these specimens, 27 were collected in Belize and comprised four species of *Ototyphlonemertes*. Ten specimens collected from sites at Bocas del Toro were representative of four genera and five species. Additionally, the Pacific Panama sites provided 18 specimens, consisting of eight morphological species included in five identified genera (Table 1). The genus *Ototyphlonemertes* exhibited the highest abundance and diversity, with 34 individuals belonging to seven morphological species. The phylogenetic reconstruction, utilizing the 18S rRNA gene for the 55 analyzed nemertean hosts, provided support for the species identifications based on morphology, as all genera and species formed monophyletic clades (Figure 2).

**TABLE 1:** List of nemertean samples used in this study with information on sampling location, taxonomic affiliation, GenBank accession numbers, geographic coordinates of sample sites, number of genetic reads retrieved with the 16S rRNA prokaryotic gene, and number of microbial ASVs.

Sample ID	Locality	Ocean	Class	Order	Family	Genus	Species	GenBank#	Longitude	Latitude	Microbial Genetic Reads	Microbial ASVs
7NemPanB04	Panama‐Naos	East Pacific	Paleonemertea	Archinemertea	Cephalothricidae	*Cephalothrix*	*Cephalothrix* sp	PQ330969	−79.531	8.916	11741	255
6NemgenCephaloA08	Bocas del Toro	West Atlantic	Paleonemertea	Archinemertea	Cephalothricidae	*Cephalothrix*	*Cephalothrix* sp	PQ330972	−82.168	9.352	20189	180
5Nem1335H04	Belize	West Atlantic	Hoplonemertea	Monostilifera	Ototyphlonemertidae	*Ototyphlonemertes*	*Ototyphlonemertes santacruzensis*	KT448348	−88.082	16.803	38489	126
7NemPanD09	Panama‐Naos	East Pacific	Paleonemertea	Archinemertea	Cephalothricidae	*Cephalothrix*	*Cephalothrix* sp	PQ330966	−79.126	8.388	9782	125
7NemPanF06	Panama‐Naos	East Pacific	Hoplonemertea	Monostilifera	NA	"Psammonemertes"*	"Psammonemertes" sp.	PQ330976	−79.554	8.801	3334	118
6NemgenHubrechtellaC05	Bocas del Toro	West Atlantic	Pilidiophora	Hubrechtiiformes	Hubrechtidae	*Hubrechtella*	*Hubrechtella* sp	PQ330965	−82.168	9.347	9916	117
7NemPanE06	Panama‐Naos	East Pacific	Hoplonemertea	Monostilifera	Ototyphlonemertidae	*Ototyphlonemertes*	*Ototyphlonemertes* sp	PQ330930	−79.061	8.673	18488	103
6NemgenOtotyphC07	Bocas del Toro	West Atlantic	Hoplonemertea	Monostilifera	Ototyphlonemertidae	*Ototyphlonemertes*	*Ototyphlonemertes erneba*	MH302963	−82.168	9.352	11164	99
5NemJLNA09	Belize	West Atlantic	Hoplonemertea	Monostilifera	Ototyphlonemertidae	*Ototyphlonemertes*	*Ototyphlonemertes santacruzensis*	KT448337	−88.077	16.802	66358	75
6NemgenAnnuloB03	Bocas del Toro	West Atlantic	Hoplonemertea	Monostilifera	Tetrastemmatidae	*Annulonemertes*	*Annulonemertes* sp	PQ330975	−82.172	9.35	3891	68
7NemPanC07	Panama‐Naos	East Pacific	Paleonemertea	Archinemertea	Cephalothricidae	*Cephalothrix*	*Cephalothrix* sp	PQ330968	−79.555	8.8	13460	66
7NemPanA01	Panama‐Naos	East Pacific	Paleonemertea	Archinemertea	Cephalothricidae	*Cephalothrix*	*Cephalothrix* sp	PQ330970	−79.531	8.916	9283	62
7NemPanD01	Panama‐Naos	East Pacific	Hoplonemertea	Monostilifera	NA	"Psammonemertes"*	"Psammonemertes" sp.	PQ330977	−79.554	8.801	1065	58
8NemgenPoseidonH04	Panama‐Achotines	East Pacific	Hoplonemertea	Monostilifera	Amphiporidae	*Poseidonemertes*	*Poseidonemertes* sp	PQ330929	−80.001	7.621	839	55
6NemgenOtotyphH03	Bocas del Toro	West Atlantic	Hoplonemertea	Monostilifera	Ototyphlonemertidae	*Ototyphlonemertes*	*Ototyphlonemertes santacruzensis*	PQ330931	−82.172	9.351	13783	54
6NemgenOtotyphA01	Bocas del Toro	West Atlantic	Hoplonemertea	Monostilifera	Ototyphlonemertidae	*Ototyphlonemertes*	*Ototyphlonemertes santacruzensis*	PQ330932	−82.3	9.458	965	53
7NemPanB05	Panama‐Naos	East Pacific	Pilidiophora	Hubrechtiiformes	Hubrechtidae	*Hubrechtella*	*Hubrechtella* sp	PQ330964	−79.531	8.916	1064	50
7NemPanA11	Panama‐Naos	East Pacific	Pilidiophora	Heteronemertea	Lineidae	*Micrura*	*Micrura* sp	PQ330962	−79.531	8.916	5348	49
7NemPanB09	Panama‐Naos	East Pacific	Hoplonemertea	Monostilifera	Ototyphlonemertidae	*Ototyphlonemertes*	*Ototyphlonemertes cirrula*	PQ330957	−79.531	8.916	2637	49
5NemJLND11	Belize	West Atlantic	Hoplonemertea	Monostilifera	Ototyphlonemertidae	*Ototyphlonemertes*	*Ototyphlonemertes santacruzensis*	PQ330937	−88.077	16.802	2088	47
7NemPanD04	Panama‐Naos	East Pacific	Hoplonemertea	Monostilifera	NA	NA	NA	PQ330958	−79.554	8.801	19965	46
7NemPanC09	Panama‐Naos	East Pacific	Paleonemertea	Archinemertea	Cephalothricidae	*Cephalothrix*	*Cephalothrix* sp	PQ330967	−79.537	8.782	2498	46
6NemgenAnnuloD02	Bocas del Toro	West Atlantic	Paleonemertea	Archinemertea	Cephalothricidae	*Cephalothrix*	*Cephalothrix* sp	PQ330973	−82.175	9.345	3235	43
6NemgenAnnuloE02	Bocas del Toro	West Atlantic	Hoplonemertea	Monostilifera	Tetrastemmatidae	*Annulonemertes*	*Annulonemertes* sp	PQ330974	−82.175	9.345	653	43
7NemPanB03	Panama‐Naos	East Pacific	Hoplonemertea	Monostilifera	Ototyphlonemertidae	*Ototyphlonemertes*	*Ototyphlonemertes duplex*	PQ330956	−79.531	8.916	716	36
7NemPanB06	Panama‐Naos	East Pacific	Pilidiophora	Heteronemertea	Lineidae	*Micrura*	*Micrura* sp	PQ330961	−79.531	8.916	1481	34
5NemJLNB02	Belize	West Atlantic	Hoplonemertea	Monostilifera	Ototyphlonemertidae	*Ototyphlonemertes*	*Ototyphlonemertes santacruzensis*	PQ330938	−88.079	16.802	1416	34
5NemJLNG01	Belize	West Atlantic	Hoplonemertea	Monostilifera	Ototyphlonemertidae	*Ototyphlonemertes*	*Ototyphlonemertes santacruzensis*	PQ330935	−88.079	16.802	3000	31
7NemPanB08	Panama‐Naos	East Pacific	Pilidiophora	Heteronemertea	Lineidae	*Micrura*	*Micrura* sp	PQ330959	−79.531	8.916	1504	31
5NemJLNF07	Belize	West Atlantic	Hoplonemertea	Monostilifera	Ototyphlonemertidae	*Ototyphlonemertes*	*Ototyphlonemertes lactea*	KT448288	−88.077	16.803	2295	29
5NemJLNE07	Belize	West Atlantic	Hoplonemertea	Monostilifera	Ototyphlonemertidae	*Ototyphlonemertes*	*Ototyphlonemertes lactea*	PQ330948	−88.077	16.803	2145	27
5NemJLNE05	Belize	West Atlantic	Hoplonemertea	Monostilifera	Ototyphlonemertidae	*Ototyphlonemertes*	*Ototyphlonemertes lactea*	PQ330949	−88.082	16.803	3253	26
7NemPanC11	Panama‐Naos	East Pacific	Pilidiophora	Hubrechtiiformes	Hubrechtidae	*Hubrechtella*	*Hubrechtella* sp	PQ330963	−79.537	8.782	887	26
5NemJLNB09	Belize	West Atlantic	Hoplonemertea	Monostilifera	Ototyphlonemertidae	*Ototyphlonemertes*	*Ototyphlonemertes macintoshi*	KT448326	−88.077	16.802	9722	23
5NemJLNE02	Belize	West Atlantic	Hoplonemertea	Monostilifera	Ototyphlonemertidae	*Ototyphlonemertes*	*Ototyphlonemertes lactea*	PQ330950	−88.078	16.804	2660	23
5NemJLND05	Belize	West Atlantic	Hoplonemertea	Monostilifera	Ototyphlonemertidae	*Ototyphlonemertes*	*Ototyphlonemertes lactea*	PQ330951	−88.082	16.803	1770	22
5NemJLNE08	Belize	West Atlantic	Hoplonemertea	Monostilifera	Ototyphlonemertidae	*Ototyphlonemertes*	*Ototyphlonemertes lactea*	PQ330947	−88.077	16.803	1082	21
5NemJLNG02	Belize	West Atlantic	Hoplonemertea	Monostilifera	Ototyphlonemertidae	*Ototyphlonemertes*	*Ototyphlonemertes lactea*	PQ330945	−88.078	16.804	1770	20
5NemJLNA02	Belize	West Atlantic	Hoplonemertea	Monostilifera	Ototyphlonemertidae	*Ototyphlonemertes*	*Ototyphlonemertes santacruzensis*	PQ330939	−88.079	16.801	2613	19
5NemJLNH01	Belize	West Atlantic	Hoplonemertea	Monostilifera	Ototyphlonemertidae	*Ototyphlonemertes*	*Ototyphlonemertes santacruzensis*	PQ330933	−88.079	16.802	557	19
5NemJLNA08	Belize	West Atlantic	Hoplonemertea	Monostilifera	Ototyphlonemertidae	*Ototyphlonemertes*	*Ototyphlonemertes lactea*	PQ330953	−88.076	16.802	1077	18
5NemJLNB08	Belize	West Atlantic	Hoplonemertea	Monostilifera	Ototyphlonemertidae	*Ototyphlonemertes*	*Ototyphlonemertes lactea*	PQ330952	−88.077	16.802	1964	16
7NemPanB07	Panama‐Naos	East Pacific	Pilidiophora	Heteronemertea	Lineidae	*Micrura*	*Micrura* sp	PQ330960	−79.531	8.916	754	14
5NemJLNF03	Belize	West Atlantic	Hoplonemertea	Monostilifera	Ototyphlonemertidae	*Ototyphlonemertes*	*Ototyphlonemertes santacruzensis*	PQ330936	−88.082	16.802	96	14
5NemJLNG03	Belize	West Atlantic	Hoplonemertea	Monostilifera	Ototyphlonemertidae	*Ototyphlonemertes*	*Ototyphlonemertes santacruzensis*	PQ330934	−88.082	16.802	385	12
5Nem1335D03	Belize	West Atlantic	Hoplonemertea	Monostilifera	Ototyphlonemertidae	*Ototyphlonemertes*	*Ototyphlonemertes santacruzensis*	PQ330940	−88.082	16.802	11076	9
5NemJLNF06	Belize	West Atlantic	Hoplonemertea	Monostilifera	Ototyphlonemertidae	*Ototyphlonemertes*	*Ototyphlonemertes lactea*	PQ330946	−88.082	16.803	1075	9
6NemgenOtotyphE08	Bocas del Toro	West Atlantic	Hoplonemertea	Monostilifera	Ototyphlonemertidae	*Ototyphlonemertes*	*Ototyphlonemertes erneba*	PQ330954	−82.168	9.352	459	9
5NemJLNE06	Belize	West Atlantic	Hoplonemertea	Monostilifera	Ototyphlonemertidae	*Ototyphlonemertes*	*Ototyphlonemertes erneba*	KT427994	−88.082	16.803	455	9
5NemJLNF08	Belize	West Atlantic	Hoplonemertea	Monostilifera	Ototyphlonemertidae	*Ototyphlonemertes*	*Ototyphlonemertes macintoshi*	PQ330942	−88.077	16.803	197	9
5NemJLND06	Belize	West Atlantic	Hoplonemertea	Monostilifera	Ototyphlonemertidae	*Ototyphlonemertes*	*Ototyphlonemertes erneba*	PQ330955	−88.082	16.803	260	7
6NemgenCephaloD07	Bocas del Toro	West Atlantic	Paleonemertea	Archinemertea	Cephalothricidae	*Cephalothrix*	*Cephalothrix* sp	PQ330971	−82.172	9.351	87	7
5NemJLND02	Belize	West Atlantic	Hoplonemertea	Monostilifera	Ototyphlonemertidae	*Ototyphlonemertes*	*Ototyphlonemertes macintoshi*	PQ330944	−88.077	16.804	657	5
5NemJLNG06	Belize	West Atlantic	Hoplonemertea	Monostilifera	Ototyphlonemertidae	*Ototyphlonemertes*	*Ototyphlonemertes macintoshi*	PQ330941	−88.082	16.803	1986	4
5NemJLNF02	Belize	West Atlantic	Hoplonemertea	Monostilifera	Ototyphlonemertidae	*Ototyphlonemertes*	*Ototyphlonemertes macintoshi*	PQ330943	−88.077	16.803	1009	2

* Distinct genus without formal description. Interstitial nemertean with an unusually elongate precerebral region and typically 4–6 eyes set in a row on each side of the head. Refer to Figure 22.1d in Norenburg JL. Interstitial Nemertina. Introduction to the Study of Meiofauna. 1988. [Correction added on 11 December 2024, after first online publication: Due to a rendering error, Table 1 has been corrected to remove duplicated text.]

A total of 1393 microbial ASVs were identified across the 55 nemertean host samples. The number of ASVs and genetic reads recovered from each host is provided in the supplemental material (Data S1 and S2). Given the variability in the prokaryotic 16S rRNA gene copy numbers both across species and within species (Větrovský and Baldrian 2013), only ASVs were considered in further phylogenetic and statistical analyses.

The 10 hosts with the highest number of associated microbial ASVs (68–255) were distributed across the three locations: Pacific Panama (*N* = 4), Bocas del Toro (*N* = 4), and Belize (*N* = 2), representing four different genera (Table 1). Among the nemertean microbiomes analyzed, the most prevalent genus was *Vibrio*, detected in 92% of the samples (Data S1 and S2; Figure S1). Notably, in a specific sample of *Ototyphlonemertes macintoshi* from Belize, all prokaryotic reads were assigned exclusively to the *Vibrio* genus. The second most frequently identified genus was *Alteromonas*, present in 88% of the samples and found across all nemertean species. Following *Alteromonas*, *Cutibacterium* was detected in 79% of the samples, although it was absent in *Ototyphlonemertes cirrula* from Pacific Panama. Other genera with notable representation included *Pseudoalteromonas* and *Pseudomonas* (both present in 66% of samples), *Marinomonas* (62%), and *Mesoflavibacter* and *Ralstonia* (both found in 55% of samples). *Streptococcus* was detected in 51% of the samples. All other genera were present in fewer than half of the samples (Data S1 and S2; Figure S1).

**FIGURE 3:**
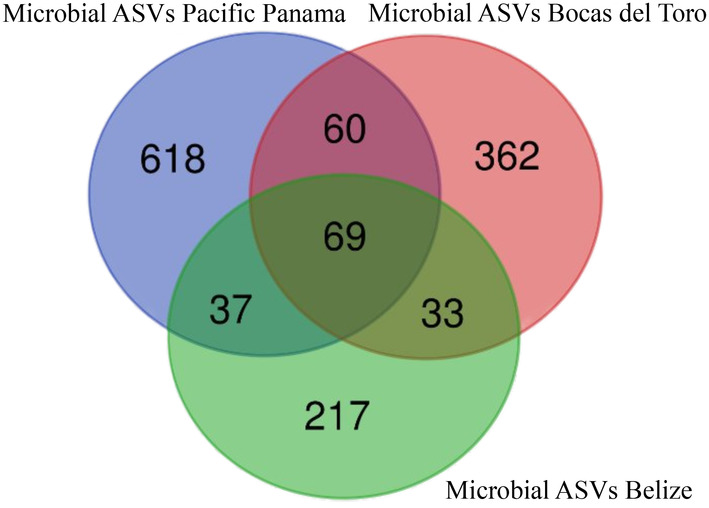
Venn diagram illustrating the number of unique and shared microbial ASVs identified from host animals collected across the three locations. The diagram highlights both location‐specific ASVs and those shared between locations.

Among the three locations where host animals were sampled, Pacific Panama had the highest overall number of microbial ASVs, with 784 ASVs across 18 host samples, followed by Bocas del Toro with 524 ASVs from 10 samples. Belize, despite having the largest number of host samples (27), recorded 356 ASVs. When examining unique microbial ASVs, only 69 out of the 1393 ASVs were shared among the three locations (Figure 3) with Pacific Panama having the highest percentage of unique ASVs (79%), followed by Bocas del Toro (69%) and Belize (61%).

**FIGURE 4:**
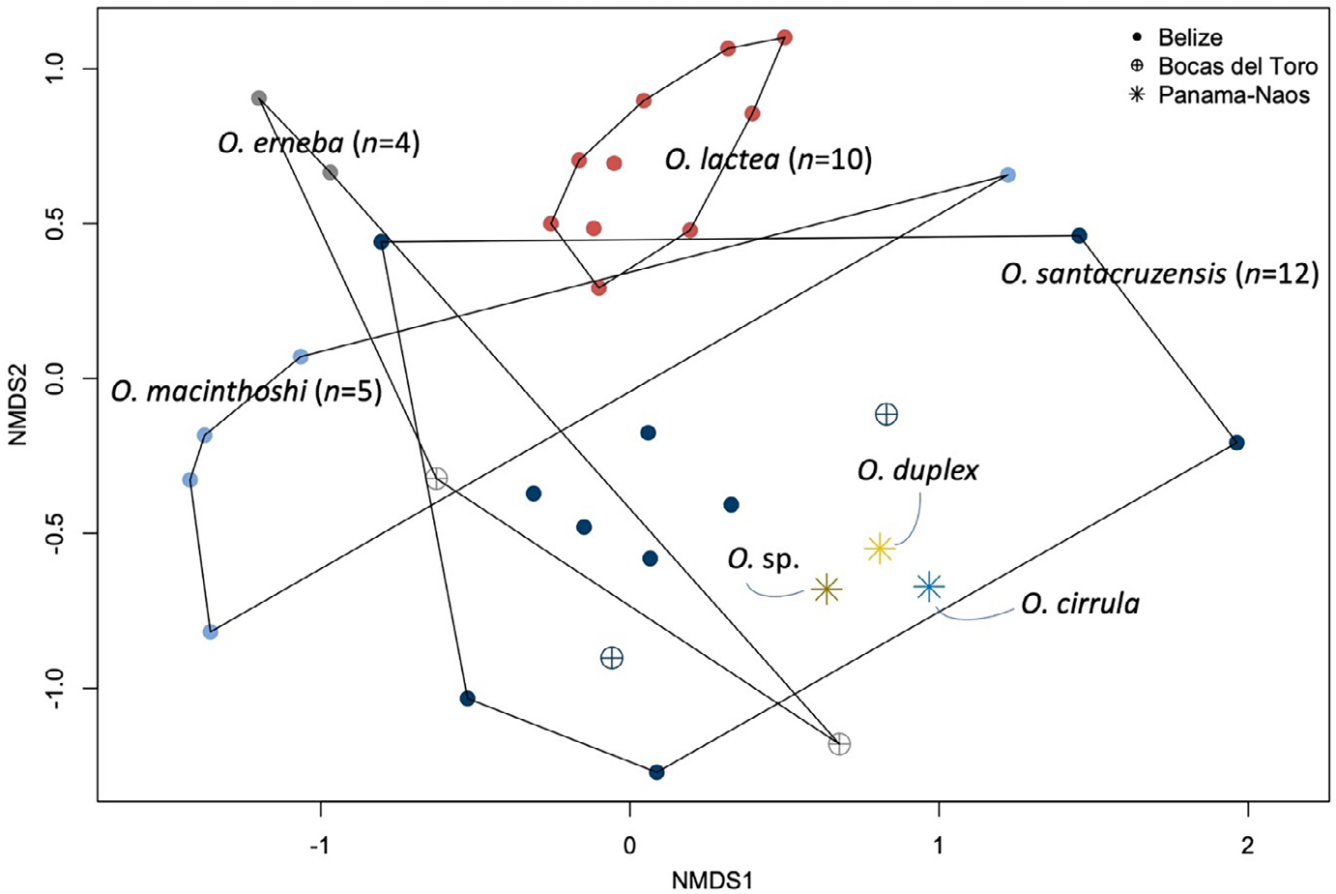
Non‐metric multidimensional scaling (nMDS) plot showing differences in microbiome composition between samples of *Ototyphlonemertes* spp. with color according to the nemertean species and shape according to the sampling locations.

Despite a high number of unique microbial ASVs identified in each location, differences in microbiome composition (Bray‐Curtis dissimilarities on ASVs from 16S rRNA gene Illumina sequencing), limited to the 54 samples identified at least to genus level (Table 1 and Data S1 and S2) were explained for 17.2% (PERMANOVA) by genus identity of the nemertean host and only for 8.0% by the geographic area of origin. A large amount of variability, 74.8%, was not explained by the taxonomic and geographic variables we included in the model. Focusing on the most densely sampled genus, *Ototyphlonemertes*, species identity explained 37.3% (PERMANOVA) of the differences in microbiome composition and geography only 4.9%, with 57.8% of unexplained variance (Figure 4).

There was no correlation between genetic distances of the hosts and differences in ASV composition of the microbiomes for the overall dataset (Mantel test: *r* = 0.054, *p* = 0.119), but the correlation was positive and significant for the dataset including only the 34 animals of the genus *Ototyphlonemertes* (*r* = 0.282, *p* = 0.001). The correlation remained significant even when controlling for geographical distances in partial Mantel tests, both with distances calculated as the crow flies (*r* = 0.281, *p* = 0.001) and along the coastline (*r* = 0.273, *p* = 0.001).

## 4 Discussion

This study provides valuable insights into the complex relationships between microbiome diversity, host genetic relatedness, and environmental factors in marine interstitial nemerteans. The principal finding highlights host genetic relatedness as a more influential factor in determining microbiome diversity than the geographical location of the host, notwithstanding a large unexplained variability in microbiome composition. The host‐microbiome correlation is evident when comparing the microbial communities among host species within a genus and not including different species of different genera in the phylum. Our findings align with those of Boscaro et al. (2022), who observed an absence of phylosymbiosis across various meiofaunal taxa at the phylum level, with a potential, though inconclusive, signal at lower taxonomic levels such as family or genus. This difficulty in detecting phylosymbiosis in natural populations may be attributed to extensive environmental variability, including microbiome plasticity, stochastic processes, and the adaptive immune system, which can obscure phylosymbiotic signals, particularly over extended evolutionary periods. For example, the detection of *Vibrio* as the sole bacterium in one of our samples could suggest an infection, potentially further obscuring the signal. To address this challenge, laboratory‐controlled studies could be beneficial to identify phylosymbiotic communities and specific microbial taxa that differentiate between host species. Unfortunately, microbiota from the surrounding aquatic environment was not investigated, which limits our understanding of the potential influence of local prokaryotes on the observed patterns in nemerteans. This omission could mean that we are missing critical data on microbial communities that inhabit the water and potentially interact with the organisms studied, influencing their microbiota. Other confounding factors could include environmental contamination. Despite our efforts to minimize contamination during both fieldwork and laboratory procedures, its occurrence remains a possibility. The challenge arises from processing whole small‐sized animals for DNA analysis, which complicates the distinction between symbiotic microbiota and external contaminants, such as prokaryotes accidentally ingested with diet, sporadic commensals, and environmental microbiota. Nonetheless, since individuals of the same species were collected from diverse locations and processed in the laboratory alongside other species, it is improbable that environmental microbiota or either systematic or sporadic contamination could account for the observed phylosymbiosis pattern.

Within the genus *Ototyphlonemertes*, species identity accounted for approximately 40% of the differences in microbiome composition among individuals. Additionally, there was a significant correlation between the phylogenetic distances of the hosts and the differences in the composition of their associated microbial communities, irrespective of geographical distances, even if with a relatively low strength of the correlation, with *r* = 0.282. Our findings align with numerous prior studies on phylosymbiosis across diverse animal taxa (Li et al. 2022; Tinker and Ottesen 2020). Furthermore, our results are consistent with previous investigations on nemertean taxa associated with toxicity, which demonstrated a strong correlation between nemertean species and their microbiomes, particularly with *Vibrio* (Mcevoy et al. 1997; Melnikova and Magarlamov 2020; Beleneva et al. 2014; Turner et al. 2018). However, the phenotypic, functional, and ecological implications of the nemertean‐microbiome relationship remain largely unknown. This necessitates mechanistic approaches, such as interspecific microbiome transplant experiments, to better understand these interactions (Brooks et al. 2016). Moreover, while phylosymbiosis is commonly linked to mutualistic interactions, vertical transmission, and coevolution, it is important to recognize that alternative mechanisms may contribute to the observed patterns. Phylosymbiosis does not inherently imply mutualistic interactions between hosts and their microbiome, and the concepts of phylosymbiosis and mutualism present distinct hypotheses that warrant empirical testing (Lim and Bordenstein 2020).

In addition to gaining insights into the functional role of host microbiota, investigating the underlying mechanisms involved in microbiota transmission within populations and across generations represents a captivating area of research and remains a prominent topic of study in human research (Funkhouser and Bordenstein 2013). Meiofaunal species exhibit characteristics that can have implications for the horizontal and/or vertical transmission of microbial organisms. For example, they exhibit a diverse array of reproductive modalities, including both sexual and asexual modes, variations in mating behavior, the potential for dormant stages, and different feeding strategies (e.g., filter feeders, detritivores, or predators) (Giere 2009; Higgins and Thiel 1988). Nemerteans can reproduce sexually and, although studies on nemertean mating behavior are limited, observations have recognized that mating interactions often involve multiple individuals prior to fertilization, with four distinct gamete transfer mechanisms: free‐spawning, mucus‐spawning, internal fertilization, and specific structures (Thiel and Junoy 2006).

The observed patterns of phylosymbiosis in nemerteans may be influenced by sexual reproduction, which facilitates both conspecific and horizontal transfer of microbiota. This reproductive strategy contrasts with that of other microscopic aquatic invertebrates, such as the predominantly parthenogenetic rotifers, in which phylosymbiosis has not been detected (Eckert, Anicic, and Fontaneto 2021; Eckert et al. 2023; Rosa and Loreto 2022; Turgay et al. 2020). Additionally, nemertean species are capable of reproducing asexually through fragmentation (Ikenaga et al. 2019; Kajihara and Hookabe 2019; Zattara et al. 2019), a process that may enable direct microbiota transfer to new individuals, offering further explanation for the observed microbial associations.

Another hypothesis that may explain phylosymbiosis in nemerteans is related to their feeding strategy. As primarily predatory organisms, nemerteans acquire microbiota that can vary based on their selected prey, which may possess distinct microbiomes. This selective acquisition through diet contrasts with that of generalist filter‐feeders like rotifers, whose microbiomes may be indiscriminately acquired from the surrounding environment through their feeding processes (Eckert, Anicic, and Fontaneto 2021; Eckert et al. 2022; Rosa and Loreto 2022; Turgay et al. 2020).

In addition to phylosymbiosis, the diversity of the microbiota in nemerteans is influenced to a lesser extent by sampling location. The nemertean species examined inhabit the benthic interstitial zone of marine environments, and the specimens were collected from distinct locations, each likely characterized by unique microhabitat features. For example, Belize and Bocas del Toro, though both situated along the Atlantic Ocean, differ considerably in their environmental contexts; Belize is uniquely sheltered by a large bay and hosts the northern hemisphere's largest barrier reef. Panama sampling sites are located on the Pacific Ocean. These geographic differences may introduce environmental variability that could influence microbiome composition. However, despite each site exhibiting a high proportion of unique ASVs, the geographic signal is notably less pronounced when assessing microbial community composition compared to the more robust patterns established by phylosymbiosis.

Yet, a significant portion of the microbiota diversity remains unaccounted for. This unexplained diversity may be influenced by microhabitat‐specific factors, such as subtle variations in microscopic chemical and physical conditions or slight differences in diet. Additionally, potential lab contamination could diminish the explained variability and enhance the unexplained variability. Stochastic factors may also play a fundamental role in shaping microbiota diversity across species, populations, and even individuals (Kohl 2020; Moeller et al. 2017; Qin et al. 2023).

In conclusion, our results demonstrated, for the first time, evidence of phylosymbiosis in meiofaunal animals like marine interstitial nemerteans. Further investigation is required to unravel the intricate mechanisms and dynamics underlying host‐microbiota associations across diverse organisms and ecological contexts. Factors such as reproductive modalities, feeding strategies, fluctuations in the local environmental conditions, and the potential influence of dormant stages are likely to contribute to the shaping of microbiota transmission patterns and host‐microbiota interactions. With little doubt, the immense diversity of meiofaunal animals, spanning over 25 animal phyla and occupying diverse ecosystems on Earth (Giere 2009; Giere and Schratzberger 2023; Higgins and Thiel 1988), presents a valuable opportunity to study the coevolutionary dynamics between hosts and their microbiota. To gain a comprehensive understanding of meiofauna‐microbiota relationships, future studies could explore the following approaches: (i) recording a wide range of environmental variables in the field, including both microbial composition and abiotic parameters; (ii) complementing fieldwork with laboratory experiments whenever feasible; and (iii) investigating phylosymbiosis at various taxonomic levels, from populations to species and higher taxonomic ranks.

## Author Contributions

**Francesca Leasi:** conceptualization (equal), data curation (equal), formal analysis (equal), funding acquisition (equal), investigation (equal), methodology (equal), project administration (equal), resources (equal), software (equal), supervision (equal), validation (equal), visualization (equal), writing – original draft (equal), writing – review and editing (equal). **Ester M. Eckert:** data curation (equal), formal analysis (equal), software (equal), validation (equal), visualization (equal), writing – original draft (supporting), writing – review and editing (equal). **Jon L. Norenburg:** data curation (supporting), funding acquisition (equal), investigation (equal), methodology (equal), resources (equal), visualization (supporting), writing – review and editing (equal). **W. Kelley Thomas:** conceptualization (equal), funding acquisition (equal), resources (equal), software (equal), writing – review and editing (equal). **Joseph L. Sevigny:** data curation (supporting), formal analysis (supporting), methodology (supporting), software (equal), validation (equal), writing – review and editing (equal). **Jeffrey A. Hall:** data curation (supporting), investigation (supporting), methodology (equal), writing – review and editing (equal). **Herman H. Wirshing:** data curation (supporting), formal analysis (equal), methodology (equal), writing – review and editing (equal). **Diego Fontaneto:** conceptualization (equal), data curation (equal), formal analysis (equal), methodology (equal), software (equal), supervision (equal), validation (equal), visualization (equal), writing – original draft (equal), writing – review and editing (equal).

## Conflicts of Interest

The authors declare no conflicts of interest.

## Supporting information


Figure S1.

**Data S1.** Microbiota community composition in nemertean specimens based on amplicon sequence variants (ASVs) and genetic read frequency. Each ASVs is identified with a unique ID (Feature ID), which is the typical output of QIIME2.

**Data S2.** Taxonomic classification of Amplicon Sequence Variants (ASVs) identified through Illumina 16S rRNA sequencing, including the number of genetic reads and sample frequency for each ASV.

## Data Availability

All data files and scripts used for the analyses can be found here: https://github.com/LeasiFrancesca/Microbiome‐Nemertea. Raw sequences of microbiota have been deposited in the NCBI SRA (Sequence Read Archive) database with SubmissionID: SUB14651484 and BioProject ID: PRJNA1161389. Accession numbers for the animal 18S rRNA genetic sequences are available in Table 1.

## References

[ece370471-bib-0001] Ahern, O. M. , K. A. Whittaker , T. C. Williams , D. E. Hunt , and T. A. Rynearson . 2021. “Host Genotype Structures the Microbiome of a Globally Dispersed Marine Phytoplankton.” Proceedings of the National Academy of Sciences 118, no. 48: e2105207118. 10.1073/pnas.2105207118 PMC8640791 34810258

[ece370471-bib-0002] Akyol, T. Y. , S. Sato , and I. Turkan . 2020. “Deploying Root Microbiome of Halophytes to Improve Salinity Tolerance of Crops.” Plant Biotechnology Reports 14, no. 2: 143–150. 10.1007/s11816-020-00594-w.

[ece370471-bib-0003] Apprill, A. , C. A. Miller , A. M. Van Cise , et al. 2020. “Marine Mammal Skin Microbiotas Are Influenced by Host Phylogeny.” Royal Society Open Science 7, no. 5: 192046. 32537203 10.1098/rsos.192046 PMC7277249

[ece370471-bib-0004] Archie, E. A. , and J. Tung . 2015. “Social Behavior and the Microbiome.” Current Opinion in Behavioral Sciences 6: 28–34.

[ece370471-bib-0005] Bletz, M. C. , H. Archer , R. N. Harris , et al. 2017. “Host Ecology Rather Than Host Phylogeny Drives Amphibian Skin Microbial Community Structure in the Biodiversity Hotspot of Madagascar.” Frontiers in Microbiology 8: 1530. 28861051 10.3389/fmicb.2017.01530 PMC5563069

[ece370471-bib-0077] Beleneva, I. A. , T. Y. Magarlamov , and A. D. Kukhlevsky . 2014. “Characterization, Identification, and Screening for Tetrodotoxin Production by Bacteria Associated with the Ribbon Worm (Nemertea) *Cephalotrix simula* (Ivata, 1952).” Microbiology 83: 220–226. 10.1134/S0026261714030059. 25844441

[ece370471-bib-0006] Bolyen, E. , J. R. Rideout , M. R. Dillon , et al. 2019. “Reproducible, Interactive, Scalable and Extensible Microbiome Data Science Using QIIME 2.” Nature Biotechnology 37, no. 8: 852–857. 10.1038/s41587-019-0209-9. PMC7015180 31341288

[ece370471-bib-0007] Boscaro, V. , C. C. Holt , N. W. L. Van Steenkiste , et al. 2022. “Microbiomes of Microscopic Marine Invertebrates Do Not Reveal Signatures of Phylosymbiosis.” Nature Microbiology 7, no. 6: 810–819. 10.1038/s41564-022-01125-9. 35618773

[ece370471-bib-0008] Brooks, A. W. , K. D. Kohl , R. M. Brucker , E. J. van Opstal , and S. R. Bordenstein . 2016. “Phylosymbiosis: Relationships and Functional Effects of Microbial Communities Across Host Evolutionary History.” PLoS Biology 14, no. 11: e2000225. 27861590 10.1371/journal.pbio.2000225 PMC5115861

[ece370471-bib-0009] Brown, B. R. P. , J. R. Goheen , S. D. Newsome , et al. 2023. “Host Phylogeny and Functional Traits Differentiate Gut Microbiomes in a Diverse Natural Community of Small Mammals.” Molecular Ecology 32: 2320–2334. 36740909 10.1111/mec.16874

[ece370471-bib-0010] Callahan, B. J. , P. J. McMurdie , M. J. Rosen , A. W. Han , A. J. A. Johnson , and S. P. Holmes . 2016. “DADA2: High‐Resolution Sample Inference From Illumina Amplicon Data.” Nature Methods 13, no. 7: 581–583. 10.1038/nmeth.3869. 27214047 PMC4927377

[ece370471-bib-0011] Caporaso, J. G. , J. Kuczynski , J. Stombaugh , et al. 2010. “QIIME Allows Analysis of High‐Throughput Community Sequencing Data.” Nature Methods 7, no. 5: 335–336. 10.1038/nmeth.f.303. 20383131 PMC3156573

[ece370471-bib-0012] Chernyshev, A. V. 2021. “An Updated Classification of the Phylum Nemertea.” Invertebrate Zoology 18: 188–196.

[ece370471-bib-0013] Conroy, T. E. , and L. Holman . 2022. “Social Immunity in the Honey Bee: Do Immune‐Challenged Workers Enter Enforced or Self‐Imposed Exile?” Behavioral Ecology and Sociobiology 76, no. 2: 32.

[ece370471-bib-0014] Dalenberg, H. , P. Maes , B. Mott , K. E. Anderson , and M. Spivak . 2020. “Propolis Envelope Promotes Beneficial Bacteria in the Honey Bee (Apis Mellifera) Mouthpart Microbiome.” Insects 11, no. 7: 453. 32708479 10.3390/insects11070453 PMC7412495

[ece370471-bib-0015] Douglas, A. E. 2018. Fundamentals of Microbiome Science: How Microbes Shape Animal Biology. Princeton, NJ: Princeton University Press.

[ece370471-bib-0016] Dunaj, S. J. , B. R. Bettencourt , J. E. Garb , and R. M. Brucker . 2020. “Spider Phylosymbiosis: Divergence of Widow Spider Species and Their tissues' Microbiomes.” BMC Evolutionary Biology 20, no. 1: 1–17. 32811423 10.1186/s12862-020-01664-x PMC7433143

[ece370471-bib-0017] Eckert, E. M. , N. Anicic , and D. Fontaneto . 2021. “Freshwater Zooplankton Microbiome Composition Is Highly Flexible and Strongly Influenced by the Environment.” Molecular Ecology 30, no. 6: 1545–1558. 10.1111/mec.15815. 33484584

[ece370471-bib-0018] Eckert, E. M. , T. Cancellario , P. L. E. Bodelier , et al. 2022. “A Combination of Host Ecology and Habitat but Not Evolutionary History Explains Differences in the Microbiomes Associated With Rotifers.” Hydrobiologia: 1–9.

[ece370471-bib-0078] Eckert, E. M. , T. Cancellario , P. L. Bodelier , et al. 2023. “A combination of host ecology and habitat but not evolutionary history explains differences in the microbiomes associated with rotifers.” Hydrobiologia 850, no. 17: 3813–3821. 10.1007/s10750-022-04958-x.

[ece370471-bib-0019] Erlandson, S. , X. Wei , J. Savage , J. Cavender‐Bares , and K. Peay . 2018. “Soil Abiotic Variables Are More Important Than Salicaceae Phylogeny or Habitat Specialization in Determining Soil Microbial Community Structure.” Molecular Ecology 27, no. 8: 2007–2024. 29603835 10.1111/mec.14576

[ece370471-bib-0020] Escalas, A. , J. C. Auguet , A. Avouac , et al. 2021. “Ecological Specialization Within a Carnivorous Fish Family Is Supported by a Herbivorous Microbiome Shaped by a Combination of Gut Traits and Specific Diet.” Frontiers in Marine Science 8: 622883. 10.3389/fmars.2021.622883.

[ece370471-bib-0021] Funkhouser, L. J. , and S. R. Bordenstein . 2013. “Mom Knows Best: The Universality of Maternal Microbial Transmission.” PLoS Biology 11, no. 8: e1001631. 23976878 10.1371/journal.pbio.1001631 PMC3747981

[ece370471-bib-0022] Giere, O. 2009. Meiobenthology: The Microscopic Motile Fauna of Aquatic Sediments. Berlin, Germany: Springer Science & Business Media.

[ece370471-bib-0023] Giere, O. , and M. Schratzberger . 2023. New Horizons in Meiobenthos Research: Profiles, Patterns and Potentials. Berlin, Germany: Springer Nature.

[ece370471-bib-0024] Gomaa, F. , D. R. Utter , W. Loo , D. J. G. Lahr , and C. M. Cavanaugh . 2022. “Exploring the Protist Microbiome: The Diversity of Bacterial Communities Associated With Arcella Spp.(Tubulina: Amoebozoa).” European Journal of Protistology 82: 125861. 35051873 10.1016/j.ejop.2021.125861

[ece370471-bib-0025] Grieneisen, L. E. , M. J. E. Charpentier , S. C. Alberts , et al. 2019. “Genes, Geology and Germs: Gut Microbiota Across a Primate Hybrid Zone Are Explained by Site Soil Properties, Not Host Species.” Proceedings of the Royal Society B 286, no. 1901: 20190431. 31014219 10.1098/rspb.2019.0431 PMC6501927

[ece370471-bib-0026] Grond, K. , K. C. Bell , J. R. Demboski , M. Santos , J. M. Sullivan , and S. M. Hird . 2020. “No Evidence for Phylosymbiosis in Western Chipmunk Species.” FEMS Microbiology Ecology 96, no. 1: fiz182. 10.1093/femsec/fiz182. 31730167

[ece370471-bib-0027] Guindon, S. , J.‐F. Dufayard , V. Lefort , M. Anisimova , W. Hordijk , and O. Gascuel . 2010. “New Algorithms and Methods to Estimate Maximum‐Likelihood Phylogenies: Assessing the Performance of PhyML 3.0.” Systematic Biology 59, no. 3: 307–321. 20525638 10.1093/sysbio/syq010

[ece370471-bib-0028] Hammer, T. J. , D. H. Janzen , W. Hallwachs , S. P. Jaffe , and N. Fierer . 2017. “Caterpillars Lack a Resident Gut Microbiome.” Proceedings of the National Academy of Sciences 114, no. 36: 9641–9646. 10.1073/pnas.1707186114 PMC5594680 28830993

[ece370471-bib-0029] Hammer, T. J. , J. G. Sanders , and N. Fierer . 2019. “Not all Animals Need a Microbiome.” FEMS Microbiology Letters 366, no. 10: fnz117. 31132110 10.1093/femsle/fnz117

[ece370471-bib-0030] Higgins, R. P. , and H. Thiel . 1988. Introduction to the Study of Meiofauna. Washington, DC: Smithsonian Institution Press.

[ece370471-bib-0031] Ikenaga, J. , N. Hookabe , H. Kohtsuka , M. Yoshida , and H. Kajihara . 2019. “A Population Without Females: Males of *Baseodiscus Delineatus* (Nemertea: Heteronemertea) Reproduce Asexually by Fragmentation.” Zoological Science 36, no. 4: 348–353. 10.2108/zs180203. 34664906

[ece370471-bib-0032] Kajihara, H. , and N. Hookabe . 2019. “Anterior regeneration in Baseodiscus hemprichii (Nemertea: Heteronemertea).” Tropical Natural History 19, no. 1: 39–42.

[ece370471-bib-0033] Katoh, K. , and D. M. Standley . 2013. “MAFFT Multiple Sequence Alignment Software Version 7: Improvements in Performance and Usability.” Molecular Biology and Evolution 30, no. 4: 772–780. 10.1093/molbev/mst010. 23329690 PMC3603318

[ece370471-bib-0034] Kohl, K. D. 2020. “Ecological and Evolutionary Mechanisms Underlying Patterns of Phylosymbiosis in Host‐Associated Microbial Communities.” Philosophical Transactions of the Royal Society B 375, no. 1798: 20190251. 10.1098/rstb.2019.0251 PMC7133527 32200746

[ece370471-bib-0035] Leasi, F. , S. C. D. S. Andrade , and J. Norenburg . 2016. “At Least Some Meiofaunal Species Are Not Everywhere. Indication of Geographic, Ecological and Geological Barriers Affecting the Dispersion of Species of Ototyphlonemertes (Nemertea, Hoplonemertea).” Molecular Ecology 25, no. 6: 1381–1397. 10.1111/mec.13568. 26840255

[ece370471-bib-0036] Leasi, F. , and L. J. Norenburg . 2014. “The Necessity of DNA Taxonomy to Reveal Cryptic Diversity and Spatial Distribution of Meiofauna, With a Focus on Nemertea.” PLoS One 9, no. 8: e104385. 10.1371/journal.pone.0104385. 25093815 PMC4122443

[ece370471-bib-0037] Leasi, F. , J. L. Sevigny , E. M. Laflamme , et al. 2018. “Biodiversity Estimates and Ecological Interpretations of Meiofaunal Communities Are Biased by the Taxonomic Approach.” Communications Biology 1, no. 1: 112. 10.1038/s42003-018-0119-2. 30271992 PMC6123632

[ece370471-bib-0038] Ley, R. E. , M. Hamady , C. Lozupone , et al. 2008. “Evolution of Mammals and Their Gut Microbes.” Science 320, no. 5883: 1647–1651. 18497261 10.1126/science.1155725 PMC2649005

[ece370471-bib-0039] Li, J. , X. Wei , D. Huang , and J. Xiao . 2022. “The Phylosymbiosis Pattern Between the Fig Wasps of the Same Genus and Their Associated Microbiota.” Frontiers in Microbiology 12: 4299. 10.3389/fmicb.2021.800190 PMC8882959 35237241

[ece370471-bib-0040] Lim, S. J. , and S. R. Bordenstein . 2020. “An Introduction to Phylosymbiosis.” Proceedings of the Royal Society B 287, no. 1922: 20192900. 32126958 10.1098/rspb.2019.2900 PMC7126058

[ece370471-bib-0041] Malešević, M. , S. Rašić , V. Santra , M. Kojić , and N. Stanisavljević . 2021. “Brevibacillus Laterosporus Supplementation Diet Modulates Honey Bee Microbiome.” Biologia Serbica 43, no. 1: 113.

[ece370471-bib-0079] Mcevoy, E. G. , A. Rogers , and R. Gibson . 1997. “Preliminary Investigation of *Vibrio alginolyticus*‐like Bacteria Associated with Marine Nemerteans.” Hydrobiologia 365: 287–291. 10.1023/A:1003174320123.

[ece370471-bib-0042] McFall‐Ngai, M. , M. G. Hadfield , T. C. G. Bosch , et al. 2013. “Animals in a Bacterial World, a New Imperative for the Life Sciences.” Proceedings of the National Academy of Sciences 110, no. 9: 3229–3236. 10.1073/pnas.1218525110. PMC3587249 23391737

[ece370471-bib-0043] Melnikova, D. I. , and T. Y. Magarlamov . 2020. “The microbial community of tetrodotoxin‐bearing and non‐tetrodotoxin‐bearing ribbon worms (Nemertea) from the Sea of Japan.” Marine Drugs 18, no. 3: 177. 10.3390/md18030177. 32210160 PMC7143766

[ece370471-bib-0044] Miller, A. J. , J. Gass , M. C. Jo , et al. 2023. “Towards the Generation of Gnotobiotic Larvae as a Tool to Investigate the Influence of the Microbiome on the Development of the Amphibian Immune System.” Philosophical Transactions of the Royal Society B 378, no. 1882: 20220125. 10.1098/rstb.2022.0125 PMC10258664 37305911

[ece370471-bib-0045] Moeller, A. H. , T. A. Suzuki , D. Lin , E. A. Lacey , S. K. Wasser , and M. W. Nachman . 2017. “Dispersal Limitation Promotes the Diversification of the Mammalian Gut Microbiota.” Proceedings of the National Academy of Sciences 114, no. 52: 13768–13773. 10.1073/pnas.1700122114 PMC5748161 29229828

[ece370471-bib-0046] Moitinho‐Silva, L. , S. Nielsen , A. Amir , et al. 2017. “The Sponge Microbiome Project.” GigaScience 6, no. 10: gix077. 29020741 10.1093/gigascience/gix077 PMC5632291

[ece370471-bib-0047] Oksanen, J. , G. L. Simpson , F. G. Blanchet , et al. 2022. “Community Ecology Package.” R Package Version 2.6–2 (2022).

[ece370471-bib-0048] Pante, E. , B. Simon‐Bouhet , J.‐O. Irisson , M. B. Simon‐Bouhet , and D. B. I. Imports . 2023. “*Package ‘marmap.’*.”

[ece370471-bib-0049] Parada, A. E. , D. M. Needham , and J. A. Fuhrman . 2016. “Every Base Matters: Assessing Small Subunit rRNA Primers for Marine Microbiomes With Mock Communities, Time Series and Global Field Samples.” Environmental Microbiology 18, no. 5: 1403–1414. 10.1111/1462-2920.13023. 26271760

[ece370471-bib-0050] Paradis, E. , and K. Schliep . 2019. “Ape 5.0: An Environment for Modern Phylogenetics and Evolutionary Analyses in R.” Bioinformatics 35, no. 3: 526–528. 30016406 10.1093/bioinformatics/bty633

[ece370471-bib-0051] Pushpakumara, B. L. D. U. , K. Tandon , A. Willis , and H. Verbruggen . 2023. “The Bacterial Microbiome of the Coral Skeleton Algal Symbiont Ostreobium Shows Preferential Associations and Signatures of Phylosymbiosis.” Microbial Ecology 86: 2032–2046. 37002423 10.1007/s00248-023-02209-7 PMC10497448

[ece370471-bib-0052] Qin, M. , L. Jiang , G. Qiao , and J. Chen . 2023. “Phylosymbiosis: The Eco‐Evolutionary Pattern of Insect–Symbiont Interactions.” International Journal of Molecular Sciences 24, no. 21: 15836. 37958817 10.3390/ijms242115836 PMC10650905

[ece370471-bib-0053] Quast, C. , E. Pruesse , P. Yilmaz , et al. 2012. “The SILVA Ribosomal RNA Gene Database Project: Improved Data Processing and Web‐Based Tools.” Nucleic Acids Research 41, no. D1: D590–D596. 23193283 10.1093/nar/gks1219 PMC3531112

[ece370471-bib-0054] R Developement Core Team . 2022. “A Language and Environment for Statistical Computing.” http://www.r‐project.org.

[ece370471-bib-0055] Rosa, M. T. , and E. L. S. Loreto . 2022. “Stenostomum Leucops (Catenulida, Platyhelminthes) has a Flexible Microbiome in Time and Space.” Hydrobiologia 850: 3675–3683. 10.1007/s10750-022-04931-8.

[ece370471-bib-0056] Röthig, T. , M. A. Ochsenkühn , A. Roik , R. van der Merwe , and C. R. Voolstra . 2016. “Long‐Term Salinity Tolerance Is Accompanied by Major Restructuring of the Coral Bacterial Microbiome.” Molecular Ecology 25, no. 6: 1308–1323. 10.1111/mec.13567. 26840035 PMC4804745

[ece370471-bib-0057] Sadeghi, J. , S. R. Chaganti , T. B. Johnson , and D. D. Heath . 2022. “Host Species and Habitat Shape Fish‐associated Bacterial Communities: Phylosymbiosis between Fish and their Microbiome.” Microbiome 11, no. 1: 258. 10.1186/s40168-023-01697-6 PMC10658978 37981701

[ece370471-bib-0058] Sands, C. J. , P. Convey , K. Linse , and S. J. McInnes . 2008. “Assessing Meiofaunal Variation Among Individuals Utilising Morphological and Molecular Approaches: An Example Using the Tardigrada.” BMC Ecology 8, no. 1: 7. 10.1186/1472-6785-8-7. 18447908 PMC2387140

[ece370471-bib-0059] Schuelke, T. , P. T. José , S. M. Hardy , and H. M. Bik . 2018. “Nematode‐Associated Microbial Taxa Do Not Correlate With Host Phylogeny, Geographic Region or Feeding Morphology in Marine Sediment Habitats.” Molecular Ecology 27, no. 8: 1930–1951. 10.1111/mec.14539. 29600535

[ece370471-bib-0060] Sweet, M. J. , and M. T. Bulling . 2017. “On the Importance of the Microbiome and Pathobiome in Coral Health and Disease.” Frontiers in Marine Science 4: 9.

[ece370471-bib-0061] Thiel, M. , and J. Junoy . 2006. “Mating Behavior of Nemerteans: Present Knowledge and Future Directions.” Journal of Natural History 40, no. 15–16: 1021–1034. 10.1080/00222930600834154.

[ece370471-bib-0062] Thomas, T. , L. Moitinho‐Silva , M. Lurgi , et al. 2016. “Diversity, Structure and Convergent Evolution of the Global Sponge Microbiome.” Nature Communications 7, no. 1: 11870. 10.1038/ncomms11870 PMC4912640 27306690

[ece370471-bib-0063] Tinker, K. A. , and E. A. Ottesen . 2020. “Phylosymbiosis Across Deeply Diverging Lineages of Omnivorous Cockroaches (Order Blattodea).” Applied and Environmental Microbiology 86, no. 7: e02513‐19. 31953337 10.1128/AEM.02513-19 PMC7082566

[ece370471-bib-0064] Turgay, E. , T. M. Steinum , K. M. Eryalçın , R. E. Yardımcı , and S. Karataş . 2020. “The Influence of Diet on the Microbiota of Live‐Feed Rotifers (Brachionus Plicatilis) Used in Commercial Fish Larviculture.” FEMS Microbiology Letters 367, no. 2: fnaa020. 10.1093/femsle/fnaa020. 32005987

[ece370471-bib-0065] Turner, A. D. , D. Fenwick , A. Powell , et al. 2018. “New Invasive Nemertean Species (Cephalothrix Simula) in England With High Levels of Tetrodotoxin and a Microbiome Linked to Toxin Metabolism.” Marine Drugs 16, no. 11: 452. 30453540 10.3390/md16110452 PMC6266807

[ece370471-bib-0066] van Oppen, M. J. H. , and L. L. Blackall . 2019. “Coral Microbiome Dynamics, Functions and Design in a Changing World.” Nature Reviews Microbiology 17, no. 9: 557–567. 31263246 10.1038/s41579-019-0223-4

[ece370471-bib-0067] Vernier, C. L. , I. M. Chin , B. Adu‐Oppong , et al. 2020. “The Gut Microbiome Defines Social Group Membership in Honey Bee Colonies.” Science Advances 6, no. 42: eabd3431. 33055169 10.1126/sciadv.abd3431 PMC7556842

[ece370471-bib-0068] Větrovský, T. , and P. Baldrian . 2013. “The Variability of the 16S rRNA Gene in Bacterial Genomes and Its Consequences for Bacterial Community Analyses.” PLoS One 8, no. 2: e57923. 23460914 10.1371/journal.pone.0057923 PMC3583900

[ece370471-bib-0069] Vilgalys, R. , and B. L. Sun . 1994. “Ancient and Recent Patterns of Geographic Speciation in the Oyster Mushroom Pleurotus Revealed by Phylogenetic Analysis of Ribosomal DNA Sequences.” Proceedings of the National Academy of Sciences of the United States of America 91, no. 10: 4599–4603. http://www.ncbi.nlm.nih.gov/pmc/articles/PMC43833/. 8183955 10.1073/pnas.91.10.4599 PMC43833

[ece370471-bib-0070] Wickham, H. , and C. Sievert . 2016. P. ggplot2: Elegant Graphics for Data Analysis, 690, 691. Berlin, Germany: Springer International.

[ece370471-bib-0071] Wickham, H. 2019. “Stringr: Simple, Consistent Wrappers for Common String Operations.” R Package Version 1.4. 0.

[ece370471-bib-0072] Wilke, C. O. , and B. Wiernik . 2022. “ggtext: Improved Text Rendering Support for ‘ggplot2.’.” R Package Version 0.1. 2.

[ece370471-bib-0073] Yeoh, Y. K. , P. G. Dennis , C. Paungfoo‐Lonhienne , et al. 2017. “Evolutionary Conservation of a Core Root Microbiome Across Plant Phyla Along a Tropical Soil Chronosequence.” Nature Communications 8, no. 1: 215. 10.1038/s41467-017-00262-8 PMC5548757 28790312

[ece370471-bib-0074] Youngblut, N. D. , G. H. Reischer , W. Walters , et al. 2019. “Host Diet and Evolutionary History Explain Different Aspects of Gut Microbiome Diversity Among Vertebrate Clades.” Nature Communications 10, no. 1: 2200. 10.1038/s41467-019-10191-3. PMC6522487 31097702

[ece370471-bib-0075] Zattara, E. E. , F. A. Fernández‐Álvarez , T. C. Hiebert , A. E. Bely , and J. L. Norenburg . 2019. “A Phylum‐Wide Survey Reveals Multiple Independent Gains of Head Regeneration in Nemertea.” Proceedings of the Royal Society B: Biological Sciences 286, no. 1898: 20182524. 10.1098/rspb.2018.2524. PMC6458331 30836873

[ece370471-bib-0076] Zilber‐Rosenberg, I. , and E. Rosenberg . 2008. “Role of Microorganisms in the Evolution of Animals and Plants: The Hologenome Theory of Evolution.” FEMS Microbiology Reviews 32, no. 5: 723–735. 18549407 10.1111/j.1574-6976.2008.00123.x

