# Supplementary material for: Microbiota Associated With Ototyphlonemertes Species (Nemertea, Hoplonemertea, Monostilifera, Ototyphlonemertidae) Reveal Evidence of Phylosymbiosis
Source: Ecol Evol. 2024 Dec 3;14(12):e70471. doi: 10.1002/ece3.70471 (PMC11612514; doi:10.1002/ece3.70471)
Supplement: Supplementary file 1 — Figure S1. [file ECE3-14-e70471-s001.pdf]

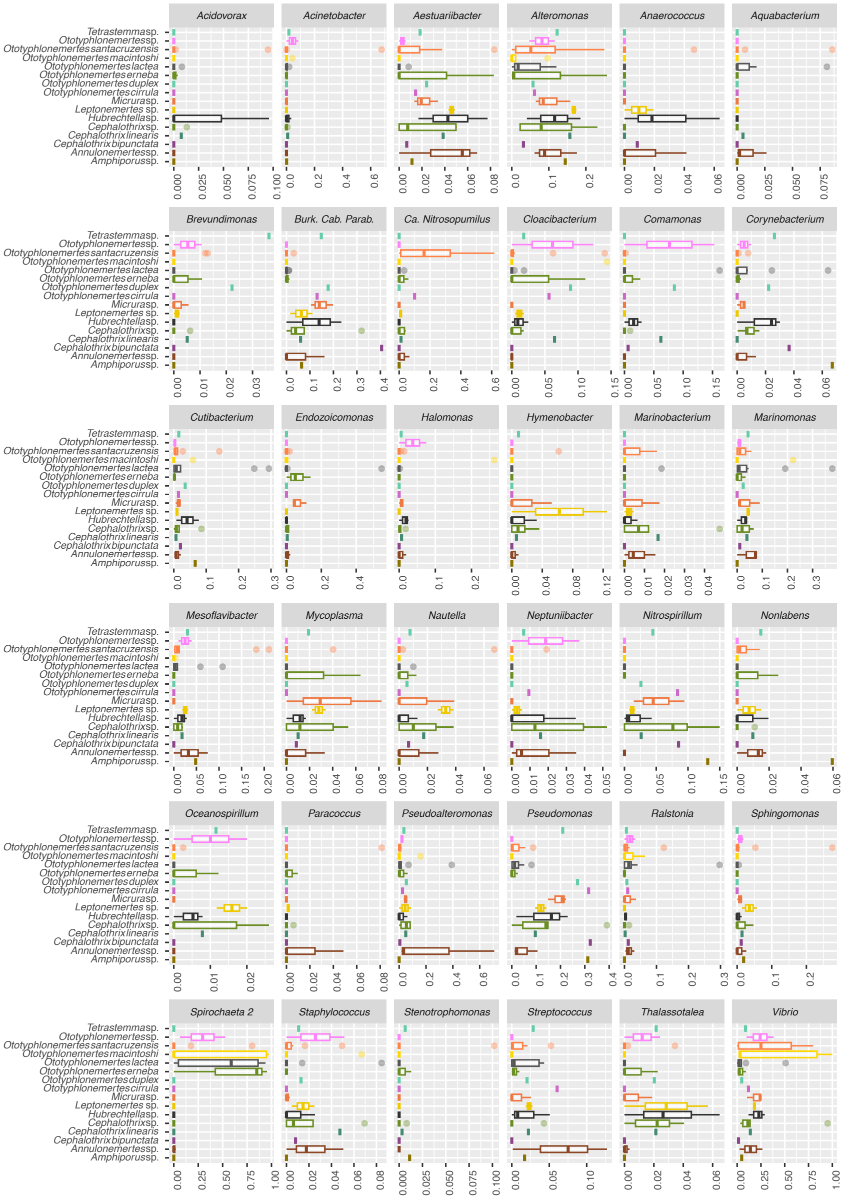

Supplemental Figure 1. Boxplots of relative abundances of the most common bacterial genera (>1000 reads) in the various species of Nemertea. The box extends from the first to the third quartile with the median denoted as a line in the middle and the whiskers indicate the variability of values with outliers denoted as dots.
